# Draft genome sequence of pathogen *Pasteurella multocida* isolated from rabbits having pneumonia in India

**DOI:** 10.1128/mra.00614-25

**Published:** 2025-10-20

**Authors:** Manjeet Chahar, Krishna Kant Sharma, Hari Mohan

**Affiliations:** 1 Centre for Medical Biotechnology, Maharshi Dayanand University 29062, Rohtak, India; 2 Department of Microbiology, Maharshi Dayanand University 29062, Rohtak, India; Rochester Institute of Technology, Rochester, New York, USA

**Keywords:** lungs, complete genome, rabbit, *Pasteurella multocida*

## Abstract

In this paper, we announce the complete genome of *Pasteurella multocida* (PMMR212) isolated from rabbits suffering from pneumonia from Rohtak, Haryana, India. The assembly contains 1,288 genes. The genome size is 2,354,118 bp (approx. 2.35 Mb).

## ANNOUNCEMENT

**Fig 1:**
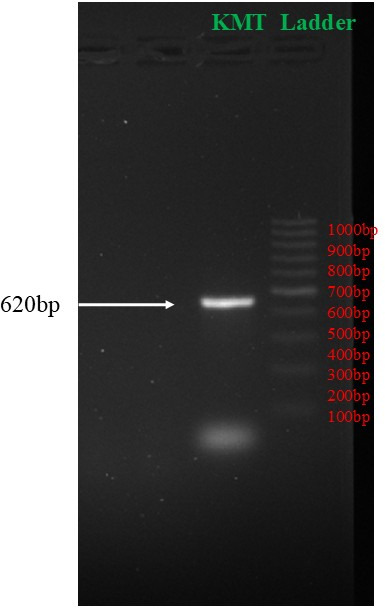
KMT band at 460bp.

PMMR212 was isolated from rabbits suffering from a pneumonia outbreak in Central Animal House, Maharshi Dayanand University, Rohtak, Haryana, India (GPS: 28.876° N, 76.615° E), in August and September 2023. *Pasteurella multocida* was isolated from the affected lungs of rabbits on brain heart infusion (BHI) agar plate at 37°C overnight. The bacteria were cultured in BHI broth for DNA isolation at 37°C overnight. DNA isolation was performed using HIMEDIA HiPurA Bacterial Genomic DNA Purification Kit according to the manufacturer’s instructions and stored at −20°C for further use. The isolated DNA was confirmed using *P. multocida*-specific primers (KMT: KMT1SP65′-GCTGTAAACGAACTCGCCAC-3′, KMT1T75′-ATCCGCTATTTACCCAGTGG-3′) and as *P. multocida* (1). Figure 1 shows KMT band 460 bp that confirms presence of *P. multocida*.

The quality of genomic DNA and double-stranded cDNA was confirmed using Qubit dsDNA estimation and agarose gel. 500 ng gDNA and 200 ng cDNA were taken forward for each sample. Library preparation was done on all samples with NEB Next Ultra II DNA library preparation kit for Illumina; Cat no: E7770 (New England Biolabs), following the manufacturer’s recommended protocol, and the sequencing was done on the S4 flow cell of NovaSeq 6000. Commercial service providers carried out library preparation and sequencing (NxGenBio Life Sciences, New Delhi, India).

DNA was delivered with ~300–400 bp insert size for every sample. The gDNA was fragmented into smaller lengths, after which it was end-repaired and A-tailed, and adapters were ligated. The products were purified and enriched through PCR to generate the final gDNA library. Library quantification was done using a Qubit Fluorometer (Invitrogen, Life Technologies, Grand Island, NY, USA). Library fragment distribution was confirmed on the HSDNA kit using TapeStation (Agilent Technologies, USA). The tagged gDNA libraries were pooled at equivalent ratios and loaded onto the c-bot automated system for cluster generation. After cluster generation, the libraries were loaded onto an Illumina S4 Flow Cell of the Illumina NovaSeq 6000 Sequencing system, and sequencing was performed using 2 × 151 bp paired-end chemistry. Following sequencing, the samples were demultiplexed, and the indexed adapter sequences were removed using the CASAVA v1.8.2 software, Illumina Inc (2). .

After adapter trimming, the Unicycler (v 0.5.1) assembles filtered reads for a *de novo* assembly. RagTag (v2.1.0) was used to scaffold *de novo* assembly using the *P. multocida* strain 10,2426 reference genome. RagTag does homology-based mis-assembly correction, homology-based assembly scaffolding, patching, and scaffold merging (3) (Table 1).

**TABLE 1:** The statistics of a genome assembly

Name	Statistics
Total_length	2354118
Number of sequences	1
Longest scaffold	2354118
Shortest scaffold	2354118
N50	2354118

## Data Availability

The bio-project has been submitted to the National Center for Biotechnology Information (NCBI), with the accession number followed by this link https://www.ncbi.nlm.nih.gov/sra/?term=PRJNA1139986. The genome sequence for P. multocida is accessible through NCBI under BioProject ID: PRJNA1139986 with SRA (Sequence read archive) link: https://ncbi.nlm.nih.gov/Traces?run=SRR29979239 .

## References

[1] Mahboob S, Ullah N, Farhan Ul Haque M, Rauf W, Iqbal M, Ali A, Rahman M. 2023. Genomic characterization and comparative genomic analysis of HS-associated Pasteurella multocida serotype B:2 strains from Pakistan. BMC Genomics 24:546. doi:10.1186/s12864-023-09626-5 37710174 PMC10500850

[2] Leggett RM, Ramir(Fig. 1)ez-Gonzalez RH, Clavijo BJ, Waite D, Davey RP. 2013. Sequencing quality assessment tools to enable data-driven informatics for high throughput genomics. Front Genet 4:288. doi:10.3389/fgene.2013.00288 24381581 PMC3865868

[3] Shi Z, Nie S, Ning S, Li F, Liu K, Huo T. 2025. RAGtag: a retrieval-augmented generation-based topic modeling framework. In Tan Y, Shi Y (ed), Data mining and big data. DMBD 2024. Communications in Computer and Information Science. Springer, Singapore. 10.1007/978-981-96-7175-5_5.

